# Supplementary figures and images for: Effects of mechanical and chemical control on invasive Spartina alterniflora in the Yellow River Delta, China
Source: PeerJ. 2019 Sep 3;7:e7655. doi: 10.7717/peerj.7655 (PMC6730535; doi:10.7717/peerj.7655)

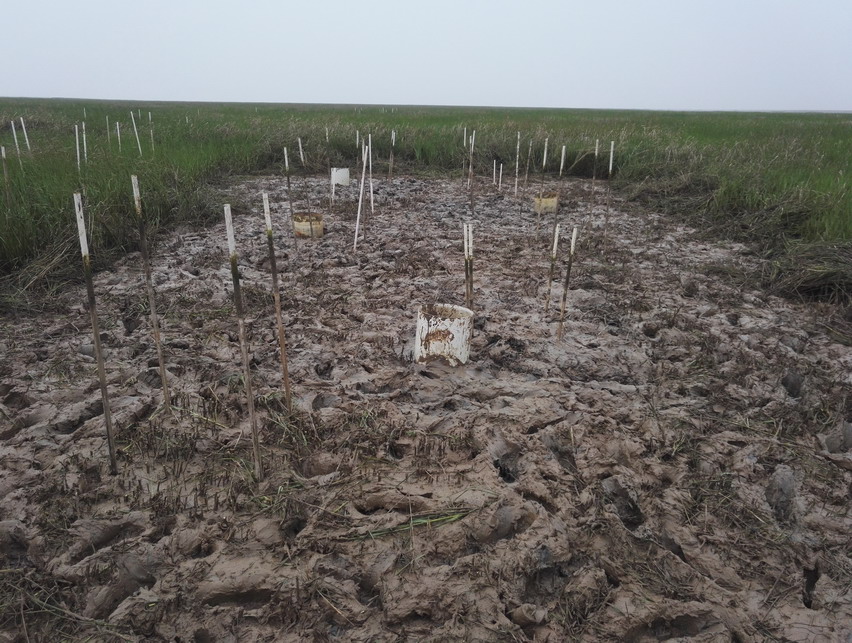

Supplement: Supplemental Information 2 [file peerj-07-7655-s002.jpg]

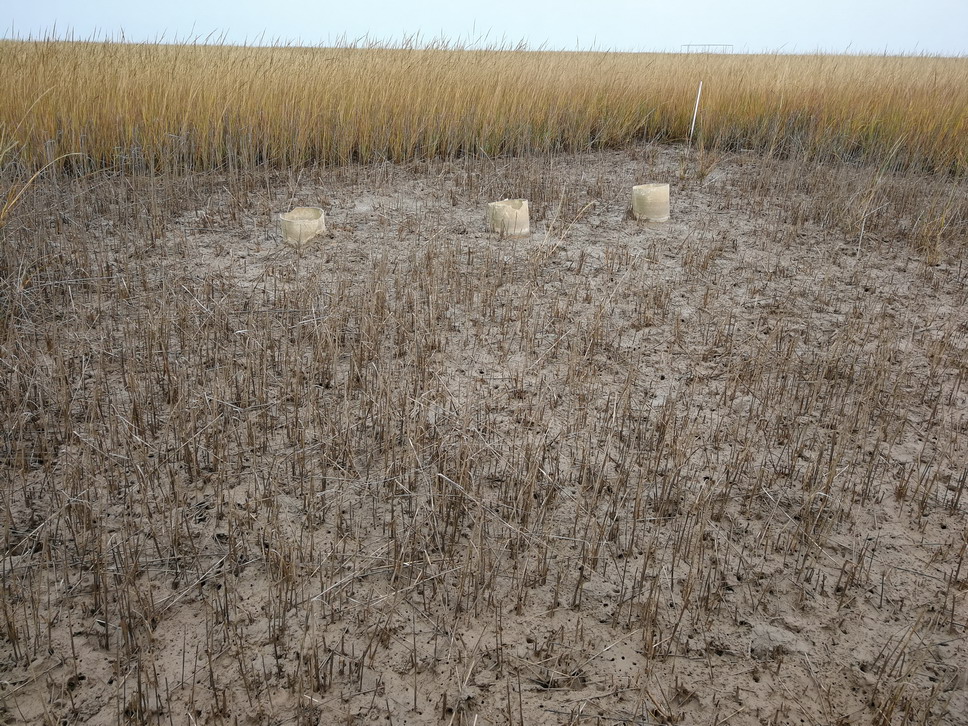

Supplement: Supplemental Information 3 [file peerj-07-7655-s003.jpg]

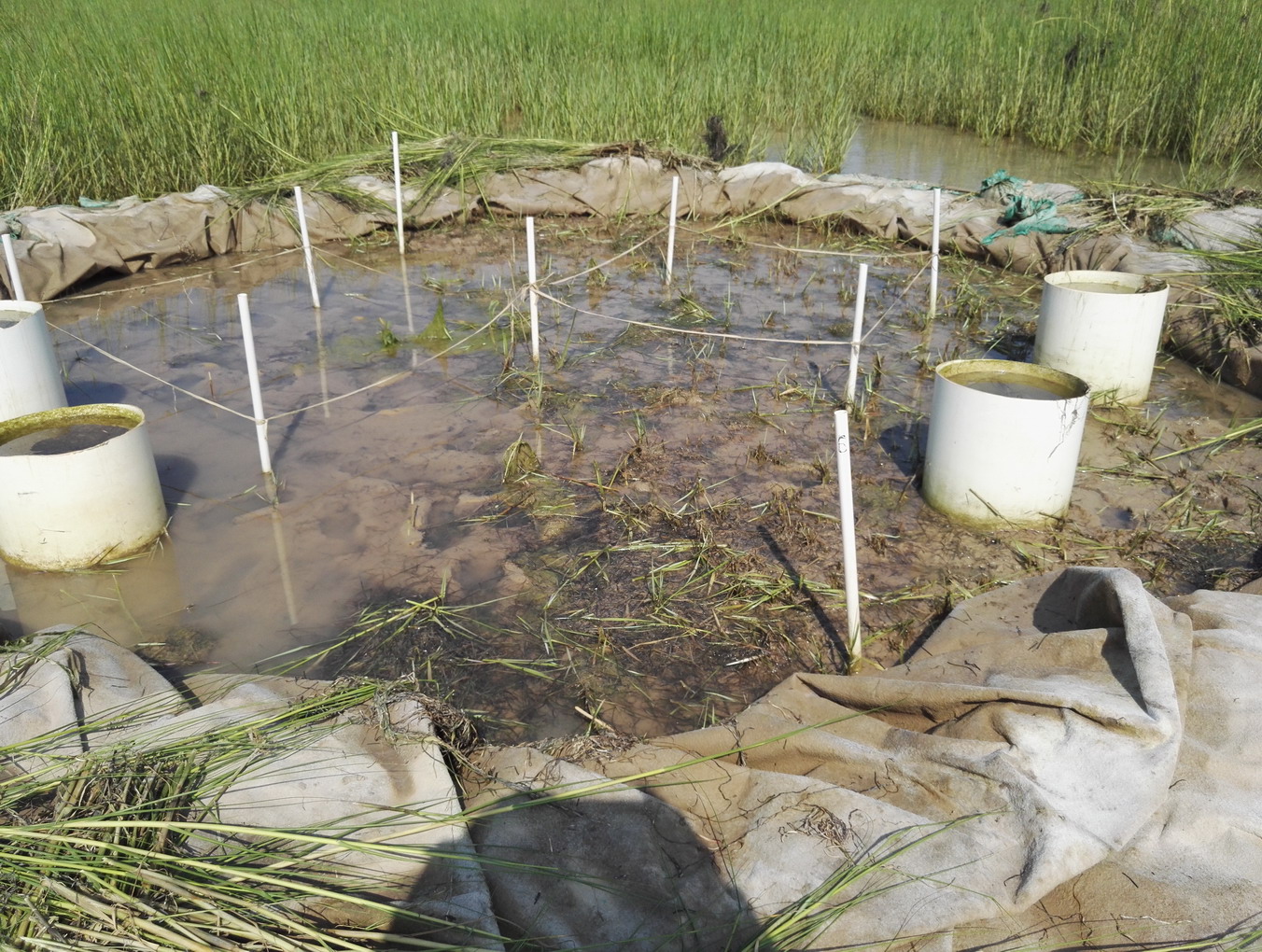

Supplement: Supplemental Information 4 [file peerj-07-7655-s004.jpg]
